# Supplementary material for: Reversible Magneto-ionic Modification of Metallic Magnetic Thin Films
Source: ACS Appl Electron Mater. 2026 Mar 27;8(7):3198–206. doi: 10.1021/acsaelm.6c00200 (PMC13085528; doi:10.1021/acsaelm.6c00200)
Supplement: Supplementary file 1 [file el6c00200_si_001.pdf]

# Supporting Information: Reversible Magneto-ionic Modification of Metallic magnetic Thin Films

Md Golam Hafiz,[*,†,‡] Hari Babu Vasili,[†] Philippa Shepley,[†] Mannan Ali,[†] Andrew J. Britton,[¶] Oscar Cespedes,[†] Weibin Li,[§] Manuel Valvidares,[§] Rohit Pachat,[‖] Wilfried Fotso,[‡] Mourad Cherif,[‡] Shimpei Ono,[⊥] Yves Roussigne,[‡] Mohamed Belmeguenai,[‡] and Gavin Burnell[*,†]

†*School of Physics and Astronomy, University of Leeds, Leeds LS2 9JT, UK*

‡*Laboratoire des Sciences des Procédés et des Matériaux, Université Paris 13 Nord, 93430 Villetaneuse, France*

¶*School of Chemical and Process Engineering, University of Leeds, Leeds LS2 9JT, UK*

§*ALBA Synchrotron Light Source, E-08290 Cerdanyola del Vallès, Barcelona, Catalonia, Spain*

‖*Centre de Nanosciences et de Nanotechnologies, CNRS, Université Paris-Saclay, 91120 Palaiseau, France*

⊥*International Center for Synchrotron Radiation Innovation Smart (SRIS), Sendai, Japan*

E-mail: hafizmdgolam927@gmail.com; g.burnell@leeds.ac.uk

## Section 1: Kerr microscopy and XMCD hysteresis loops

The Kerr microscope in Figure S1 shows the local measurement of the gated region. Figure S1 (a–b) illustrates the effect of applied voltage on the Ir/Pt and Cu/Pt samples, respectively.

Similar to the Hall hysteresis loop, both Ir/Pt and Cu/Pt maintain full remanence. Under an applied voltage of -2.1 V, the coercive field ($H_c$) increases, and with +2.1 V, it decreases. Figure S2 (a) presents the XMCD hysteresis loops of the Ir/Pt sample, while Figure S2 (b) shows the hysteresis loop of the Cu/Pt sample. The $H_c$ of the negative voltage sample is significantly increased compared to the as-grown sample.

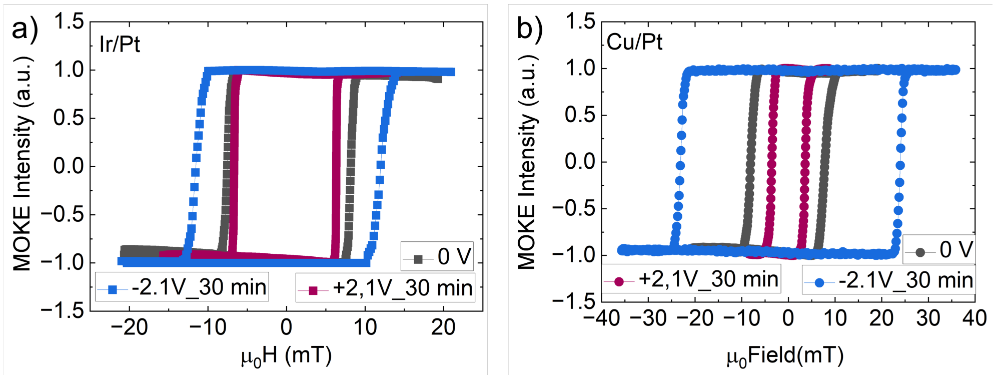


Figure S1: Kerr microscope hysteresis loops: a negative voltage is increasing $H_c$ while a positive voltage is decreasing it. (a) The hysteresis loops of the Ir/Pt top layer: initial state, with an applied negative voltage, and with an applied positive voltage. (b) The hysteresis loops of the Cu/Pt top layer: initial state, with an applied negative voltage, and with an applied positive voltage. The effect on Cu/Pt samples is also the same as for Ir/Pt, though it is more pronounced here.

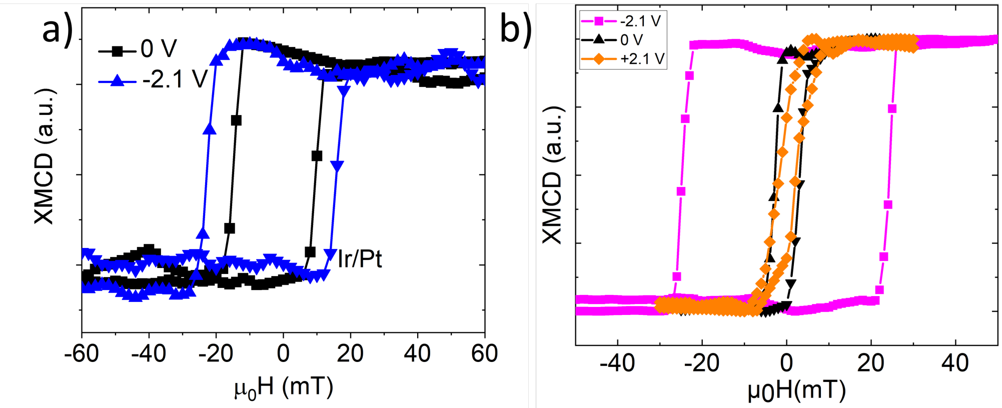


Figure S2: XMCD hysteresis loops: (a) Ir/Pt samples (b) Cu/Pt samples. For both samples the $H_c$ is increasing with a negative voltage.

# Section 2: Toy model

We employ a simple toy model to estimate the change in oxidation as a result of gating in our samples. Using the magnetic moment per unit area versus nominal thickness data (Figure 1 c), we treat the sample as consisting of a slab of CoB with a bulk magnetisation and a slab of magnetically dead oxidised Co. The as-grown data set gives a good linear fit and establishes the base-line magnetisation and dead layer thicknesses. For a sample that has been gated we allow the effective thickness of the sample to be a free parameter constrained to be between 0 and its nominal thickness plus the base-line dead layer thickness. We then perform a linear fit to the complete data set (un-gated and gated samples) and find a gradient and intercept and finally perform a least-squares regression to find the change between nominal and effective thickness of the gated sample with respect to the linear gradient and intercept compared to the base-line of the ungated data set alone.

Figure S3 shows the result of the least squares regression. For convenience we express the effective thickness of the gated sample as a shift from its nominal thickness and the goodness of fit parameter that we minimiuse is given by:

$$\mathrm{P(shift)} = \left[\left(\frac{\mathrm{m_0 - m_i}}{\mathrm{m_0}}\right)^2 + \left(\frac{\mathrm{c_0 - c_i}}{\mathrm{c_0}}\right)^2\right]^{1/2} \tag{1}$$

Where $m_0$ and $c_0$ are the magnetization thickness and the thickness of the dead layer of the as grown samples only and $m_i$ and $c_i$ are the magnetization thickness and the dead layer when including the gated sample, assuming a shifted effective thickness of the gated sample. The model gives us a $0.9 \pm 0.1$Å increase in the dead layer for the negative voltage and a $0.6 \pm 0.1$Å reduction in the dead layer thickness for the positive voltage.

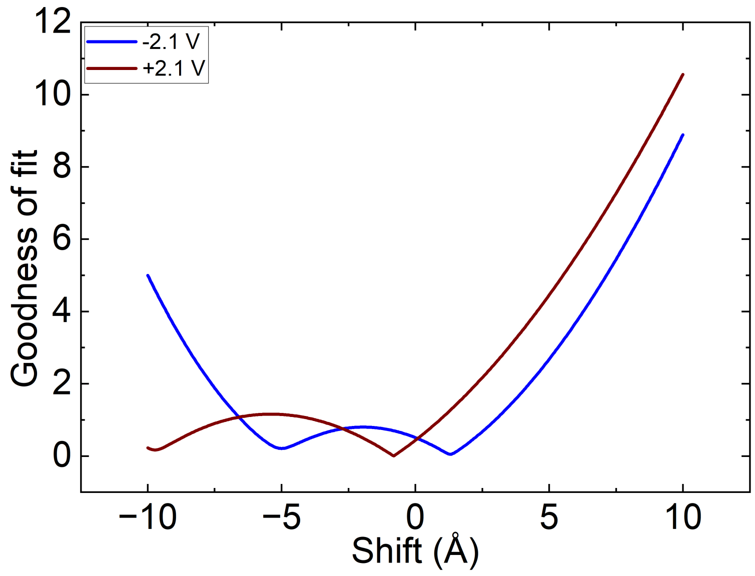


Figure S3: The goodness of fit using the toy model is evaluated by comparing the dataset including gated samples with variable effective thickness to the original linear fit of the as-grown samples alone.

## Section 3: XPS analysis

The Co 2p spectra was measured with a monochromated Al K-alpha source (1486.7 eV) in UHV (1x10-9 mbar) with a Phoibos 150 hemispherical analyser with a pass energy of 30 eV and a step size of 0.1 eV. The binding energy was calibrated using the Fermi level.

Figure S4 shows the Co $2p_{3/2}$ and Co $2p_{1/2}$ XPS data at 0 V (a and d), -2.1 V (b and e), and +2.1 V (c and f). For simplicity, only Co $2p_{3/2}$ data were fitted. The peaks at higher binding energy correspond to the Co $2p_{1/2}$ component. The as-grown Ir/Pt sample (0 V) exhibits two peaks at 778 eV and 781.5 eV. In contrast, the as-grown Cu/Pt sample displays three major peaks at 778 eV, 781 eV, and 785 eV, along with an additional small peak at 776 eV. The peak at 778 eV corresponds to the Co metal peak[1–5] . A reduction in the intensity of this peak indicates an increase in CoO, while an increase in the Co peak suggests the reduction of CoO. The oxide and satellite peaks overlap; however, the peak at 781 eV is attributed to CoO, while the peak at 785 eV is identified as a satellite peak. The satellite peaks are significantly higher than expected for standard Co references, likely due to inelastic scattering from the overlayer. The small peak at 776 eV, slightly above the

background, adjacent to the primary Co peak and has been identified as the Co Auger peak (LLM type)[6,7] .

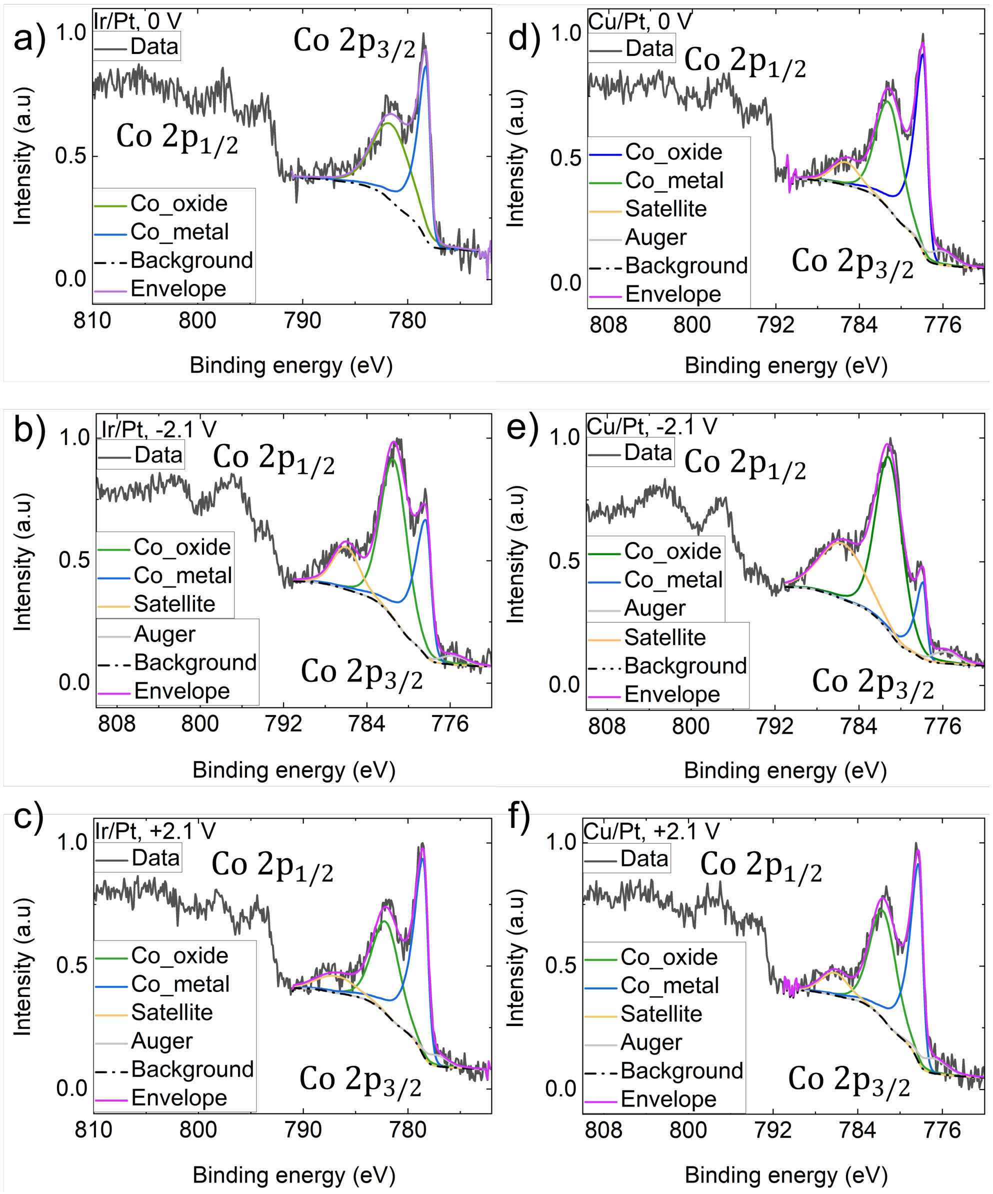


Figure S4: XPS spectra at the Co $2p_{3/2}$ and Co $2p_{1/2}$ edge: (a-c) As-grown and voltage-applied Ir/Pt samples, and (d-f) As-grown and voltage-applied Cu/Pt samples. All samples exhibit a complex Co peak, comprising metallic Co and CoO. As-grown and positively gated samples suppress the CoO peak, while negatively gated samples suppress the metallic Co peak.

# Section 4: DMI and anisotropy calculations

The DMI is calculated using,

$$\Delta \mathrm{F} = \mathrm{F_S} - \mathrm{F_{AS}} = \frac{2\gamma}{\pi \mathrm{M_s}} \mathrm{D_{eff}} \mathrm{k_{sw}} = \frac{2\gamma}{\pi} \mathrm{k_{sw}} \frac{\mathrm{D_s}}{\mathrm{t_{FM}} \mathrm{M_s}}, \tag{2}$$

where, $\Delta F$ is the frequency difference, $F_S$ is the stock frequency, $F_{AS}$ is the anti-stock frequency, $\mathrm{k_{sw}} = 4\pi \sin\theta/\lambda$ represents the spin wave vector, $\gamma$ stands for the gyromagnetic ratio, $\mathrm{D_{eff}}$ stands for the effective DMI, and $\mathrm{t_{FM}}$ represents the thickness of the magnetic layer.

The anisotropy constant is calculated from Equation,

$$H_k = \frac{2K_{eff}}{M_s} = H \frac{1 - \sqrt{2A_2}}{\sqrt{2A_2}}, \tag{3}$$

where, $\mathrm{H_k}$ is the effective anisotropy field and $\mathrm{M_s}$ is the saturation magnetization, measured using SQUID-VSM, and H is the applied field, $\mathrm{A_2}$ is the quadratic parameter of parabola[8] .

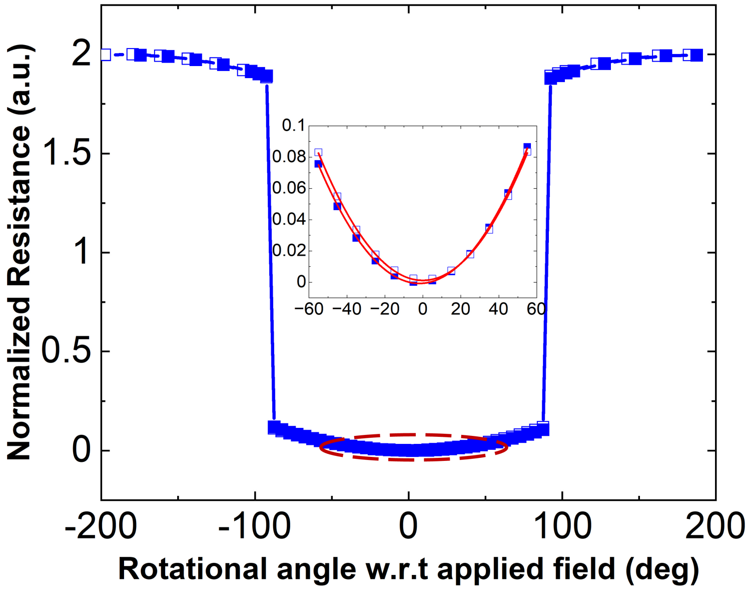


Figure S5: An example of $\mathrm{H_K}$ calculation from an AHE measurement. The normalized resistance is plotted as a function of the rotational angle relative to the applied field. The inset highlights the parabolic region and its fitting, used to determine $\mathrm{H_K}$.

# Section 5: Reversibility cycles and $R_{xx}$

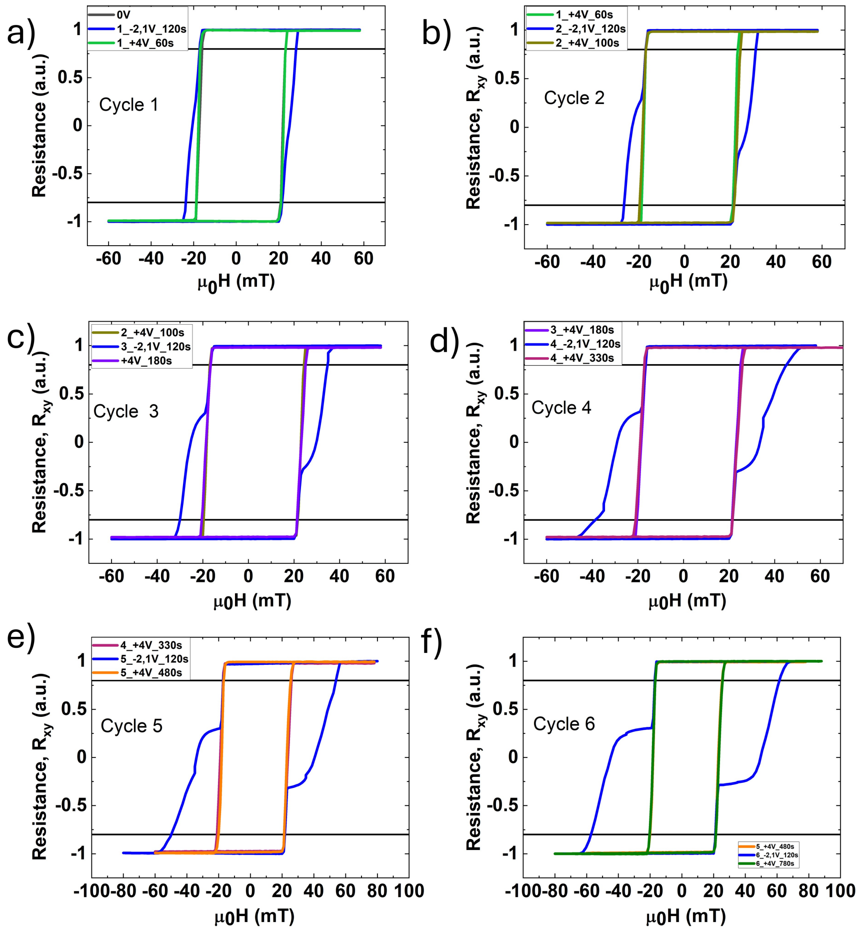


Figure S6: Reversibility cycles (a–f): The reversibility is calculated with respect to the previous cycle. A negative voltage is applied for 120 s in each cycle. The system studied in this work is almost fully reversible; the observed discrepancy may arise from slight movement of the ITO glass electrode. In each cycle, the negative-voltage response shows differences compared with the previous cycle. This behavior may be attributed to oxygen ions migrating over larger distances in each cycle.

In the sheet thin film geometry the resistance measured contains contributions from both $R_{xy}$ and $R_{xx}$. To separate the two, we decompose the data into symmetric and antisymmetric parts with respect to the magnetic field taking into account the hysteresis present and assume that the $R_{xy}$ component arises from the antisymmetric components whilst $R_{xx}$ comes from symmetric.

$$\mathrm{R_{xy}} = \frac{R_{up}(+H) - R_{down}(-H)}{2}, \tag{4}$$

$$\mathrm{R_{xx}} = \frac{R_{up}(+H) + R_{down}(-H)}{2}, \tag{5}$$

where $R_{up}(+H)$ and $R_{down}(-H)$ correspond to the resistance values at opposite signs of the applied magnetic field for the branches of the hysteresis loop sweeping from negative to positive field and from positive to negative field, respectively. Figure S7 illustrates the raw resistance measured and the decomposition into $\mathrm{R_{xy}}$ and $\mathrm{R_{xx}}$. Due to the small range of $\mathrm{R_{xx}}(\Delta \mathrm{R_{xx}}/\mathrm{R_{xx}} \approx 0.03\%)$, any hysteresis in it is obscured by numerical errors in the regions where the AHE is switching and only data for sweeping from the extrema to zero field is shown in panel c.

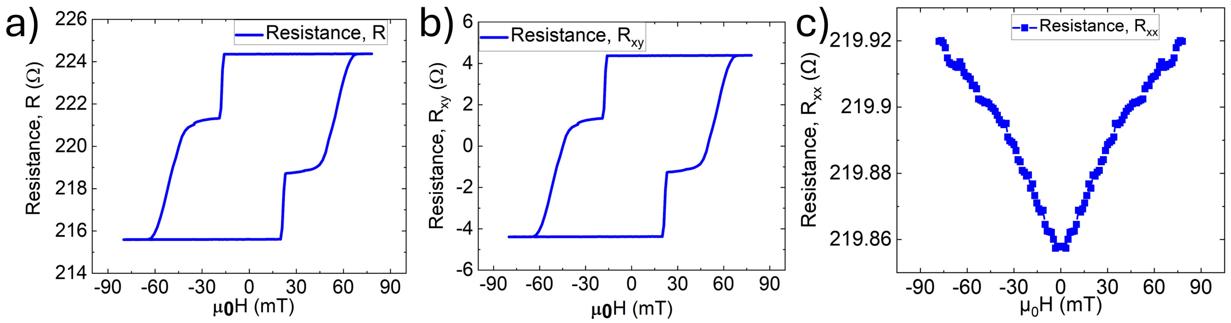


Figure S7: An example resistance of It/Pt sample: (a) Resistance, (b) $\mathrm{R_{xy}}$(c) $\mathrm{R_{xx}}$.

# Section 6: XAS signals of Ir and Pt

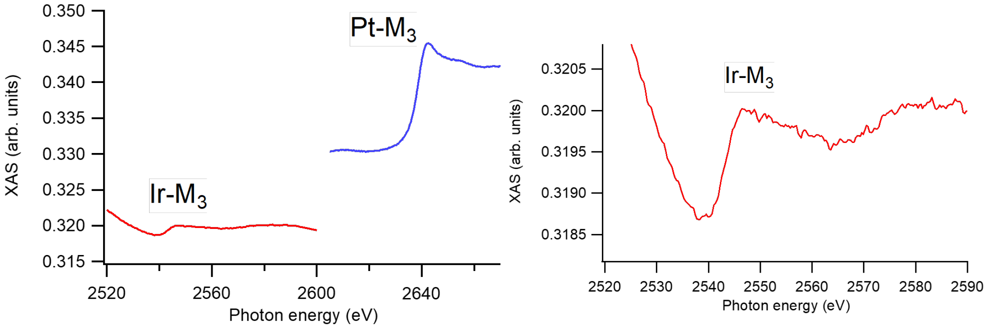


Figure S8: (left) XAS signals at Ir-M and Pt-M absorption edges. (right) XAS of Ir-M edges

# Section 7: Non-volatility effect

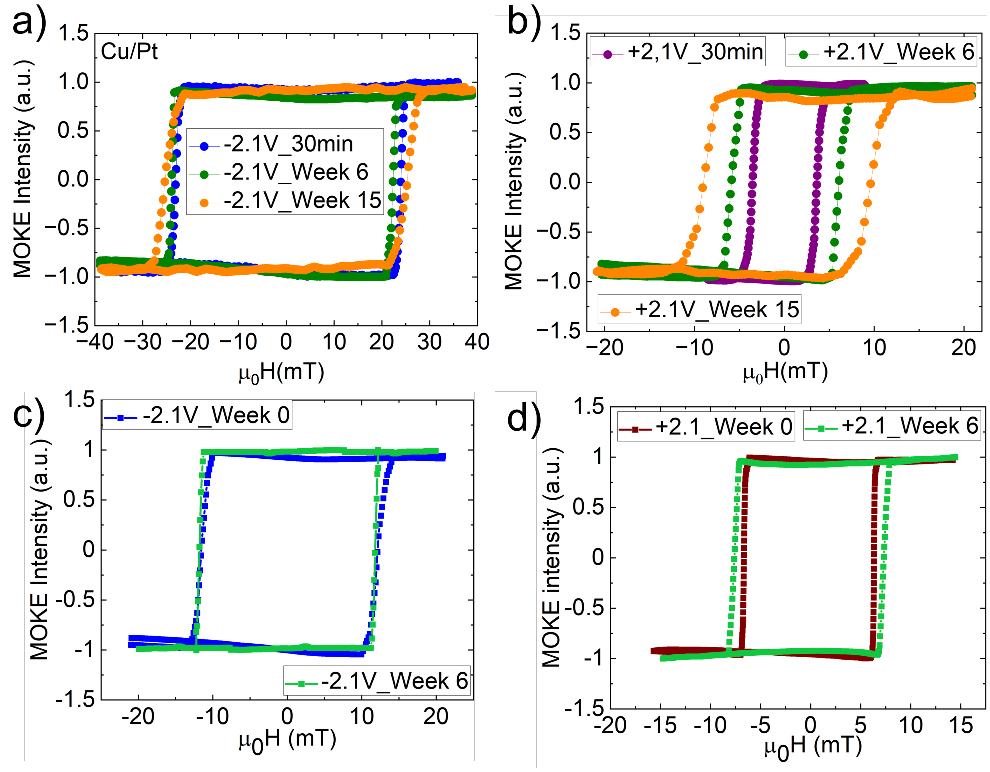


Figure S9: Non-volatility of the gating effect: (a-b) Cu/Pt samples (c-d) Ir/Pt samples

# References


(1) Grosvenor, A. P.; Wik, S. D.; Cavell, R. G.; Mar, A. Examination of the bonding in binary transition-metal monophosphides MP (M = Cr, Mn, Fe, Co) by X-ray photoelectron spectroscopy. *Inorganic Chemistry* **2005**, *44*, 8988.

(2) Yang, J.; Liu, H.; Martens, W. N.; Frost, R. L. Synthesis and characterization of Cobalt hydroxide, cobalt oxyhydroxide, and cobalt oxide nanodiscs. *Journal of Physical Chemistry C* **2010**, *114*, 111–119.

(3) Zhao, Y.; Luo, M.; Chu, S.; Peng, M.; Liu, B.; Wu, Q.; Liu, P.; de Groot, F. M.; Tan, Y. 3D nanoporous iridium-based alloy microwires for efficient oxygen evolution in acidic media. *Nano Energy* **2019**, *59*, 146–153.

(4) Feng, J.; Grimaldi, E.; Avci, C. O.; Baumgartner, M.; Cossu, G.; Rossi, A.; Gambardella, P. Effects of Oxidation of Top and Bottom Interfaces on the Electric, Magnetic, and Spin-Orbit Torque Properties of Pt / Co / Al Ox Trilayers. *Physical Review Applied* **2020**, *13*, 044029.

(5) Zhang, Z.; Li, Z.; Meng, K.; Wu, Y.; Chen, J.; Xu, X.; Jiang, Y. Perpendicular magnetic anisotropy in SrTiO3/Co/Pt films induced by oxygen diffusion from CaTiO3 spacer layer. *Applied Physics Letters* **2020**, *116*, 232402.

(6) Farr, N. G.; Griesser, H. J. XPS excitation dependence of measured cobalt 2p3 2 peak intensity due to auger peak interference. *Journal of Electron Spectroscopy and Related Phenomena* **1989**, *49*, 293–302.

(7) González-Elipe, A. R.; Yubero, F. In *Handbook of Surfaces and Interfaces of Materials*; Nalwa, H. S., Ed.; Academic Press, 2001; Vol. 2; Chapter 4, pp 147–194.

(8) Moon, K. W.; Lee, J. C.; Choe, S. B.; Shin, K. H. Determination of perpendicular

magnetic anisotropy in ultrathin ferromagnetic films by extraordinary Hall voltage measurement. *Review of Scientific Instruments* **2009**, *80*, 113904.
